# A Role for *Smoothened* during Murine Lens and Cornea Development

**DOI:** 10.1371/journal.pone.0108037

**Published:** 2014-09-30

**Authors:** Janet J. Y. Choi, Chao-Tung Ting, Lidia Trogrlic, Stefan V. Milevski, Mary Familari, Gemma Martinez, Robb U de Iongh

**Affiliations:** 1 Ocular Development Laboratory, Anatomy & Neuroscience, University of Melbourne, Parkville, Australia; 2 Department of Zoology, University of Melbourne, Parkville, Australia; Washington University, United States of America

## Abstract

Various studies suggest that Hedgehog (Hh) signalling plays roles in human and zebrafish ocular development. Recent studies (Kerr et al., *Invest Ophthalmol Vis Sci*. 2012; 53, 3316–30) showed that conditionally activating Hh signals promotes murine lens epithelial cell proliferation and disrupts fibre differentiation. In this study we examined the expression of the Hh pathway and the requirement for the *Smoothened* gene in murine lens development. Expression of Hh pathway components in developing lens was examined by RT-PCR, immunofluorescence and *in situ* hybridisation. The requirement of *Smo* in lens development was determined by conditional loss-of-function mutations, using LeCre and MLR10 Cre transgenic mice. The phenotype of mutant mice was examined by immunofluorescence for various markers of cell cycle, lens and cornea differentiation. Hh pathway components (*Ptch1, Smo, Gli2, Gli3*) were detected in lens epithelium from E12.5. Gli2 was particularly localised to mitotic nuclei and, at E13.5, Gli3 exhibited a shift from cytosol to nucleus, suggesting distinct roles for these transcription factors. Conditional deletion of *Smo*, from ∼E12.5 (MLR10 Cre) did not affect ocular development, whereas deletion from ∼E9.5 (LeCre) resulted in lens and corneal defects from E14.5. Mutant lenses were smaller and showed normal expression of p57Kip2, c-Maf, E-cadherin and Pax6, reduced expression of FoxE3 and Ptch1 and decreased nuclear Hes1. There was normal G1-S phase but decreased G2-M phase transition at E16.5 and epithelial cell death from E14.5-E16.5. Mutant corneas were thicker due to aberrant migration of Nrp2^+^ cells from the extraocular mesenchyme, resulting in delayed corneal endothelial but normal epithelial differentiation. These results indicate the Hh pathway is required during a discrete period (E9.5–E12.5) in lens development to regulate lens epithelial cell proliferation, survival and FoxE3 expression. Defective corneal development occurs secondary to defects in lens and appears to be due to defective migration of peri-ocular Nrp2^+^ neural crest/mesenchymal cells.

## Introduction

Vertebrate eye development involves co-ordinated interactions and signalling mechanisms between neural and surface ectoderm and periocular mesenchyme, which is derived from the neural crest (NC) and paraxial mesoderm. Following eye field formation, the ventral forebrain neuroepithelium evaginates laterally to form two outpocketings, the optic vesicles, which grow laterally towards the surface ectoderm. Upon contact with the surface ectoderm, the optic vesicle thickens to form the retinal disc and the pre-lens ectoderm thickens to form the lens placode. Both the lens placode and optic vesicle invaginate to form respectively the lens vesicle and the optic cup, which later gives rise to retina, iris and ciliary body. The lens vesicle subsequently differentiates to form an anterior epithelial monolayer and a mass of differentiated and elongated fibre cells [1]. The lens continues to interact with the overlying ectoderm, which subsequently differentiates to form the corneal epithelium. The corneal stroma and endothelium arise from the migration of cranial NC and mesodermal cells into the stroma that lies between the lens and overlying presumptive corneal epithelium [2], [3]. Signals from the lens have been implicated in regulating the migration of peri-ocular NC cells [4], with TGFβ2 proposed to be chemo-attractive [5], [6], while Sema3A was proposed as a chemo-repellant, based on studies in chick embryos [7].

Various growth factor families such as fibroblast growth factors (FGF), platelet-derived growth factors (PDGFs), insulin-like growth factors (IGFs), transforming growth factor-β (TGFβ) family members and Wnts have been shown to play key roles in cell proliferation or differentiation during lens development [1], [8], [9]. While significant progress has been made in understanding the roles of these growth factor signalling pathways in lens development, the Hedgehog (Hh) pathway has, until recently, not received as much attention.

Mammals and birds harbour three different Hh homologues in their genomes (Sonic, *Shh*; Desert, *Dhh*; and Indian, *Ihh*), whereas fish have six (*Shh, Dhh, Ihh* plus Tiggywinkle, *Twhh*; Echidna, *Ehh*; and Qiqihar, *Qhh*). In the absence of Hh ligand, the Patched receptors (*Ptch1* or *Ptch2*) inhibit the Smoothened 7-pass transmembrane protein (*Smo*). In the cytosol a complex of microtubule-associated proteins (Sufu, Kif7, PKA, GSK3β, CK1) function to sequester the Gli transcription factors (Gli1, Gli2, Gli3) and mediate their phosphorylation and processing to cleaved repressor forms. However in the presence of Hh ligand, the Patched-mediated inhibition of Smoothened is relieved and, by an unknown mechanism, Smoothened inhibits the Sufu-Gli complexes. This permits full length Gli proteins to enter the nucleus, where they are converted to their activator forms [10]. Gli1 and Gli2 can act as both activators and repressors whereas Gli3 acts predominantly as a repressor [11], [12] and it is the ratio of the combined activator: repressor activities that determines context-dependent cell responses [13].

There is growing evidence that Hh signalling plays important roles at various stages of eye development. Previous studies indicated that mutations in human Sonic Hedgehog (*SHH*) caused holoprosencephaly, a developmental defect involving the forebrain and midface, which in severe cases includes anophthalmia and cyclopia [14], [15]. Subsequent studies in mice [16] showed that deletion of murine *Shh* resulted in limb abnormalities, axial neural tube defects and cyclopia, and that Shh was required for separation of the eye field into bilateral domains. While Pax6 expression was still detected in these early cyclopic eyes, subsequent facial and eye development was disrupted.

Studies in zebrafish [17], [18], chick [19] and Xenopus [20] embryos have shown that inhibition of Shh or misexpression of Shh orthologues (*Twhh, Shh*) in the eye can lead to lens developmental defects as well changes in fate along the proximo-distal axis of the retina, which in many cases were accompanied by changes in expression of the paired homeobox genes, Pax6 and Pax2 [17]. Moreover, studies of the *talpid^3^* chick mutant, which is due to a mutation in the centrosomal gene, KIAA0586, resulting in failure of primary ciliogeneisis and abnormal Gli3 cleavage [21], [22], demonstrated abnormal lens development and ectopic lenses, as well as limb and craniofacial defects [21], [23] Similarly in Xenopus, modulating Shh activity by knock-down or over-expression of the antagonist, hedgehog interacting protein (Hip), resulted in abnormal eye development, including lens defects [24]. Shh signalling has also been implicated in the regeneration of a lens from the dorsal surface of the iris in the adult newt, with inhibition of Shh signalling by a cyclopamine analogue or hedgehog interacting protein (HIP) abrogating lens regeneration [25]. In zebrafish, inhibition of Hh signalling due to a truncation mutation of *Gli2* in the *you-too* (*yot*) [26], [27] or mutations of *smoothened* in the *smu* mutant [28], [29] results in ectopic lens formation due to trans-differentiation of the anterior pituitary primordium into lens structures.

Components of the Hh pathway are expressed in human lenses [30] and studies of patients with Gorlin syndrome, also known as basal cell nevus syndrome (BCNS; OMIM #109400), due to mutations in *PTCH1*, show that ∼26% have ophthalmic abnormalities, including Peter's anomaly, cataract [31]–[33] and microphthalmia [34]. While the lens phenotype of Ptch1-null mice has not been reported, mice lacking *Cdon*, which acts as a co-receptor for *Ptch1*, have abnormal lens development with decreased epithelial cell proliferation and increased apoptosis. More recently, a conditional activating mutation of the *Smo* gene in the mouse lens has been shown to result in increased cell proliferation, inhibition of fibre differentiation and aberrant induction of the epithelial marker *FoxE3* [35].

Thus there is accumulating evidence for Hh signalling having an important role in lens development. In this study we investigated the requirement for Hedgehog signalling in the rodent lens. Using conditional Cre-LoxP-mediated deletions in mice, we show that *Smo* is required during a discrete period of lens differentiation. Loss of *Smo* from ∼E13 (with the MLR10 Cre strain) had no detectable effects whereas, loss of *Smo* from ∼E10 (with the LeCre strain) results in abnormal lens development, characterized by compromised epithelial survival, abnormal cell cycle, particularly progression through the G2-M phase transition, reduced expression of FoxE3 and reduced nuclear translocation of Hes1. An additional phenotype in these eyes was aberrant development of the cornea, which appears to be due to an indirect effect of the mutant lens on migrating NC cells to the presumptive corneal stroma and endothelium.

## Materials and Methods

All animal procedures were approved by the University of Melbourne Animal Ethics Committee (Permit ID#: 1011808.1) and were carried out in accordance with the Association for Research in Vision and Ophthalmology (ARVO) statement for the Use of Animals in Ophthalmic and Vision Research. Animals were housed in standard mouse cages with a 12 hour light/dark cycle and provided environmental enrichment in their cages as well access to standard mouse chow and water *ad libitum*. Intra-peritoneal injections of mice were conducted using 26-gauge hypodermic syringes and a maximum volume of 300 µl. All animals used for experiments were killed humanely and quickly by cervical dislocation (weanling and adult mice) or decapitation (neonates and embryos).

### Generation of conditional *Smo* mutant mice

The generation of mice harbouring the LoxP-flanked *Smo* allele [36] and the LeCre or MLR10 Cre transgenes [37], [38] have been described previously. In the *Smo*-*LoxP* (*Smo^tm2Amc^*) mice (Jackson Laboratories Stock # 004526), loxP sites flank the first exon of the *Smo* gene and recombination by Cre recombinase results in a null allele. LeCre mice, on a predominantly FVB/N background, have a Cre transgene, driven by the Pax6 P0 promoter and express Cre recombinase in lens and corneal ectoderm from E9.5 onwards [37]. MLR10 mice, on a predominantly FVB/N background, harbour a Cre transgene, driven by the αA crystallin promoter in tandem with the Pax6 lens enhancer, and express Cre recombinase in the developing lens (fibres and epithelium) from E12.5 onwards [38]. In several years of breeding both Cre lines in our colonies, we have not seen any ocular phenotypes in these mice. *Smo^tm2Amc^* mice (on a C57Bl6/J background) were mated with Cre transgenic mice to generate the following genotypes, *Smo^fl/fl^/LeCre^+/−^* (hereafter referred to as LeSmox) and *Smo^fl/fl^/Mlr10^+/−^* (hereafter referred to as Smox10). Wild-type embryos used for comparison are Smo*^fl/fl^* or Smo*^fl/fl^*/LeCre^−/−^ or BCBF1 mice. Mice were genotyped by PCR analysis of genomic DNA isolated from tails or embryonic tissues using primers shown in Table 1.

**Table 1:** Genotyping Primers.

Allele	Primer	Sequence	Size (bp)	Ta (°C)
*Smo Wt*	Smo-Loxp-1	5′-CCACTGCGAGCCTTTGCGCTAC-3′	160 bp	60
	Smo-Loxp-2	5′-CCCATCACCTCCGCGTCGCA-3′		
*Smo-floxed*	Smo-Loxp-3	5′-CTTGGGTGGAGAGGCTATTC-3′	280 bp	60
	Smo-Loxp-4	5′-AGGTGAGATGACAGGAGATC-3′		
*MLR10-Cre*	PR4	5′-GCATTCCAGCTGCTGACGGT-3′	557 bp	58
	Cre-As	5′-CAGCCCGGACCGACGATGAAG		
*LeCre*	Cre-F	5′-TGACCGTACACCAAAATTTG-3′	991 bp	55
	Cre-R	5′-ATTGCCCCTGTTTCACTATC-3′		

### Reverse-Transcriptase PCR

Total RNA was collected from postnatal (P) lenses to detect expression of *Smo*, *Ptch*, and *Gli1/2/3*. Whole lenses, dissected from P3 mice, and separated into epithelial/capsule and fibre preparations, dissected from P10 mouse lenses, were stored in RNAlater (Qiagen Pty Ltd., Doncaster, VIC, Australia). Total RNA was extracted using the RNeasy Kit (Qiagen Pty Ltd., Doncaster, VIC, Australia), utilizing an on-column DNase I digestion as per manufacturer's instructions. RNA concentration and quality were measured using a Nanodrop microspectrophotometer (ThermoFisher Scientific, Wilmington, DE) and by gel electrophoresis. Total RNA (1 µg) was reverse-transcribed using the SuperScript III First-Strand Synthesis System (Invitrogen, Mulgrave, Australia) or using the Qiagen RT^2^ First Strand Kit (Qiagen) according to manufacturer's instructions. cDNA samples were amplified by PCR using primers specific for *Ptch1*, *Ptch2*, *Smo*, *Gli1/2/3* and *Hprt* (Table 2). For all experiments a minimum of three lenses were pooled per sample and at least three samples were studied.

**Table 2:** RT-PCR Primers.

Gene	Forward (5′- 3′)	Reverse (5′- 3′)	Size (bp)	Ta (°C)
*Ptch1*	TAATGCTGCGACAACTCAGG	GGCTGGAGTCTGAGAACTGG	674	50
*Ptch2*	CTTGACTGCTTCTGGGAAGG	GCCAGCATAAGCAGATAGCC	656	55
*Smo*-Exon1	CTGGGAGTCGGTTTTAATGG	ACACGTTGTAGCGCAAAGG	588	55
*Smo*-Exon4	TTGTGCTCATCACCTTCAGC	TTGAGGTCAAAAGCCAAACC	439	60
*Gli1*	CGGAGTTCAGTCAAATTAAC	CATCTGAGGTTGGGAATCC	205	67
*Gli2*	AGCCTTCACCCACCTTCTTG	TGGGCGCAGGCCCTCAGC	210	55.5
*Gli3*	CCTTCTGAGTCCTCACAGAG	GACTAGGGTTGTTCCTTCCG	158	60
*Hprt*	GCGATGATGAACCAGGTTA	GTTGAGAGATCATCTCCACC	340	53
*Smo*-Exon1-T7	TCAGAAAT**TAATACGACTCACTATAGG** CCACCTGGGAGTCGGTTTTAATGG	TCAGAAAT**TAATACGACTCACTATAGG** CCACACGTTTGTAGCGCAAAGG	619	55

### Histology and immunofluorescence

Ocular tissues from wild-type and *Smo* mutant mice and embryos were processed either for routine paraffin histology or for cryosectioning. Tissues for paraffin sectioning were fixed in 10% neutral buffered formalin (NBF) overnight at room temperature and subsequently washed in 70% ethanol before being embedded in paraffin, sectioned (∼5 µm) and mounted on positively charged slides (Superfrost Plus, Lomb Scientific, Taren Point, NSW, Australia). For cryosectioning, dissections were performed in ice-cold phosphate-buffered saline (PBS) and tissues were fixed in ice-cold 4% paraformaldehyde in PBS for 1 hour. Following rinses in PBS, tissues were cryoprotected in increasing sucrose solutions (10–30% in PBS), embedded in OCT compound (Tissue-Tek, ProScitech) and frozen in isopentane cooled by liquid nitrogen.

Paraffin sections were stained by standard haematoxylin and eosin (H&E) or immunofluorescence. For immunofluorescence, paraffin sections were re-hydrated to phosphate-buffered saline (PBS) and subjected to acid citrate-mediated antigen retrieval as described previously [39], [40]. Frozen sections and rehydrated paraffin sections were then blocked with 1% or 3% sheep serum and 0.1% bovine serum albumin in PBS for 20 min at room temperature. For experiments involving primary antibodies originating from goat, 30% CAS block (Zymed, San Francisco) with 0.1% Triton-X100 in PBS was used to block non-specific staining. Primary antibodies were applied in the respective blocking buffer overnight at 4°C using concentrations (Table 3) determined empirically. In experiments localising nuclear proteins, 0.5% Triton or 0.05% Tween-20 was included in antibody diluent. In each experiment, non-immune IgG from either rabbit or mouse at similar concentration to the primary antibody was used as negative control on one section on each slide. Nuclei in sections were stained during washes with 1 µg/ml of Hoechst dye (bisbenzimide H33258, Millipore, Billerica MA). Antibody reactivity was visualized with the appropriate AlexaFluor-conjugated secondary antibody, diluted 1∶500 in PBS with 0.1% BSA.

**Table 3:** Antibodies used for Immunofluorescence.

Polyclonal Antibodies	Cat #	Dilution	Species of Origin	Source
Anti-mouse AlexaFluor 488	A11001	1∶500	Goat	Invitrogen
Anti-rabbit AlexaFluor 488	A-11008	1∶500	Goat	Invitrogen
Anti-rabbit AlexaFluor 594	A-11037	1∶500	Goat	Invitrogen
Cyclin D1	AB21699	1∶1 Neat hybridoma fluid	Rabbit	AbCam
E-Cadherin	610182	1∶200	Mouse	BD Biosciences
FoxE3	-	1∶1000	Rabbit	Gift, Prof Peter Carlsson
Gli2	ab7181	1∶200	Rabbit	AbCam
Gli3	ab6050	1∶100	Rabbit	AbCam
Hes1	-	1∶500	Rabbit	Gift, Dr Nadean Brown
Keratin-12	sc-17101	1∶400	Goat	Santa Cruz Biotechnology, San Diego, USA
Keratin-14	ab7800	1∶200	Mouse	Abcam, Cambridge,UK
Nrp2	D39A5-3366	1∶200	Rabbit	Cell Signalling Technology,
Patched 1	ab39266	1∶200	Rabbit	AbCam
Phospho-histone-3	06-570	1∶1000	Rabbit	Upstate, Lake Placid, NY, USA
β-crystallin (3H9.3)	-	1∶1 Neat hybridoma fluid	Mouse	Gift, Prof R.C. Augusteyn

### *In situ* hybridisation

To generate *in situ* hybridization probes, PCR primers for exon 1 of the murine *Smo* gene (NM176996) were modified to include T7 RNA polymerase binding sequences (Table 2) and amplified using Pfx50 DNA polymerase (Life Technologies). The resulting PCR fragment was isolated from 1% agarose gels (Qiagen agarose gel extraction kit) and re-suspended in RNase-free water (200 ng/µl) for transcription of DIG-labelled sense and antisense RNA probes, using T7 RNA polymerase (Promega), according to manufacturer's instructions. *In situ* hybridization was performed on formalin-fixed paraffin sections using DIG-labelled riboprobes as described previously [41]–[43]. Briefly, re-hydrated sections were treated with proteinase K (20 ug/ml in 10 mM Tris pH 8, 1 mM EDTA) for 7 minutes at 37°C, before acetylation with 0.0025% acetic anhydride in 0.1 M triethanolamine for 10 minutes at room temperature. Sections were hybridized (58–60°C) overnight with DIG-labelled sense or antisense transcripts (200 ng/ml) and washed with increasing stringency (4X to 0.1X SSC at 60°C) over a period of 1.5 h. Hybridization signal was visualized by incubation with anti-DIG antibody (1∶1000) conjugated to alkaline phosphatase (AP) and histochemical detection with NBT/BCIP (Roche Diagnostics, Castle Hill, NSW Australia), according to the manufacturer's instructions.

### Image Analysis

Images were captured using either a Zeiss Axioskop II microscope, equipped with epifluorescence and a Zeiss HC camera linked to Axioviosion Imaging software (Carl Zeiss, Melbourne, VIC, Australia), or an LSM confocal microscope (Carl Zeiss). To quantify cell numbers and corneal thickness, the counting and measurement features of Axiovision (version 4.8) were used on images captured using the 40x objective. For all quantitative analyses a minimum of three sagittal sections of eyes from 4 animals were studied and at least 10 measurements were obtained per section. All data were expressed as mean ± SEM and analysed statistically using one-way ANOVA and Student's *t*-test.

## Results

### Expression of the Hedgehog (Hh) signalling pathway in lens

**Figure 1:**
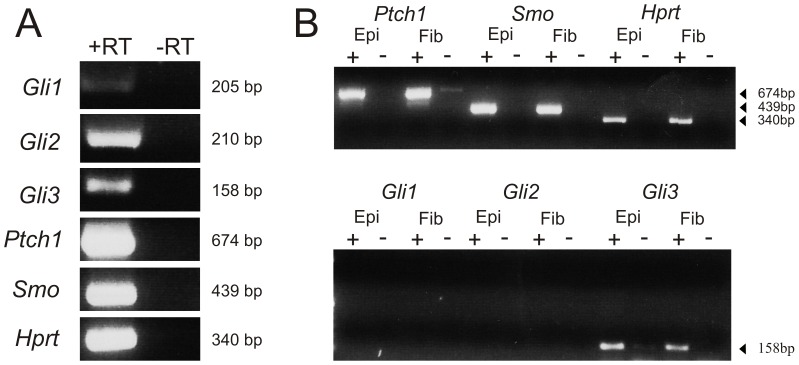
Expression of Hh pathway in lens. **A.** RT-PCR of RNA isolated from P3 lenses showing expression of *Gli1, Gli2, Gli3*, *Ptch1*, and *Smo*. Amplification of *Hprt* was included as a control house-keeping gene. **B.** RT-PCR of RNA from P10 lenses, separated into lens epithelial (Epi) and fibre (Fib) preparations, showing expression of *Ptch1*, *Smo* and *Gli3*, but not *Gli1* or *Gli2*. Presence (+) or absence (−) of reverse transcriptase in the reactions is indicated above each lane and size of each amplicon to the right of each gel.

To investigate if the developing lens expresses components of the Hh signalling pathway we carried out RT-PCR using specific primers for *Gli1-3, Ptch1*, and *Smo* on cDNA from whole neonatal lenses (P3) and P10 mouse lenses dissected into epithelial and fibre cell preparations. In whole P3 lenses, robust amplification for *Gli2, Gli3, Ptch1* and *Smo* were detected but only weak amplification of Gli1 (Fig. 1A). In P10 lenses, robust amplification of *Ptch1, Smo* and *Gli3* were detected in both epithelia and fibres but no amplification of *Gli1* or *Gli2* (Fig. 1B).

**Figure 2:**
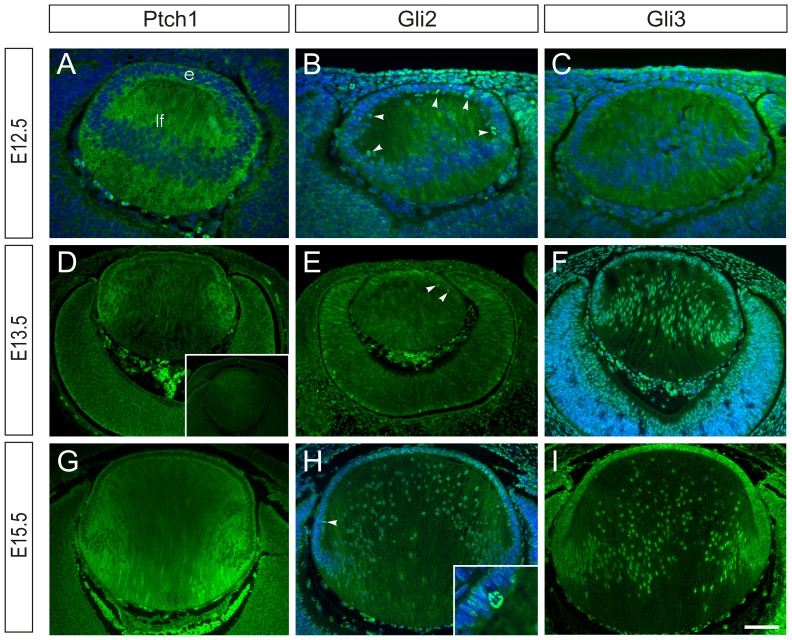
Localization of Hh pathway in lens. Immunolocalization of Ptch1 (**A, D, G**), Gli2 (**B, E, H**) and Gli3 (**C, F, I**) and non-immune IgG control (inset **D**), in developing lens at E12.5 (**A–C**), E13.5 (**D–F**) and E15.5 (**G–I**). In the lens vesicle at E12.5 cytoplasmic and membrane reactivity for Ptch1 is found in both epithelial (e) and primary lens fibre (lf) cells (**A**). Similar patterns are seen at E13.5 (**D**) and E15.5 (**G**), with reduced reactivity present in the more differentiated fibres. Predominantly nuclear reactivity for Gli2 is detected at all ages in the epithelial cells with some nuclear and cytoplasmic reactivity detected in fibre cells (**B, E, H**). Intense reactivity for Gli2 was associated with mitotic nuclei (arrowheads **B, E, H**; inset **H**). Inset in H shows a metaphase nucleus, with intense reactivity for Gli2, in the epithelium of an E15.5 lens. Diffuse cytoplasmic reactivity for Gli3 was detected in the lens vesicle at E12.5 (**C**) but distinct nuclear Gli3 reactivity was detected in epithelial and fibre cells at E13.5 and E15.5 (**F, I**). Hoechst dye fluorescence (blue) in **A–C, F** and **H** show presence of nuclei. Scale bar, 50 µm (**A–C**); 100 µm (**D–I**), 230 um (inset D), 25 µm (inset, **H**).

Confirmatory studies with antibodies to Ptch-1, Gli2 and Gli3 showed distinct expression of all three proteins from E12.5–E15.5 in the developing lens (Fig. 2). Ptch-1 was detected as cytoplasmic reactivity in the epithelium and fibre cells at all ages and appeared to be most strongly expressed in the equatorial region by E15.5 (Fig. 2D). By contrast, Gli2 was predominantly localized to nuclei of the epithelial cells and was most strongly detected in mitotic nuclei (Fig. 2B, E, H inset). At E13.5 and E15.5, Gli2 reactivity was strongest in epithelial cell nuclei but at E15.5, reactivity was also detected in the fibre cell nuclei (Fig. 2H). Gli3 reactivity was only weakly detected in the cytoplasm of E12.5 lens vesicle cells but at E13.5 and E15.5 distinct reactivity was detected in the epithelial and fibre cell nuclei (Fig. 2F, I). No staining was observed when the antibodies were replaced with similar concentrations of non-immune IgG (Fig. 2D, inset).

### Loss of *Smo* causes microphthalmia in LeSmox but not Smox10 mice

**Figure 3:**
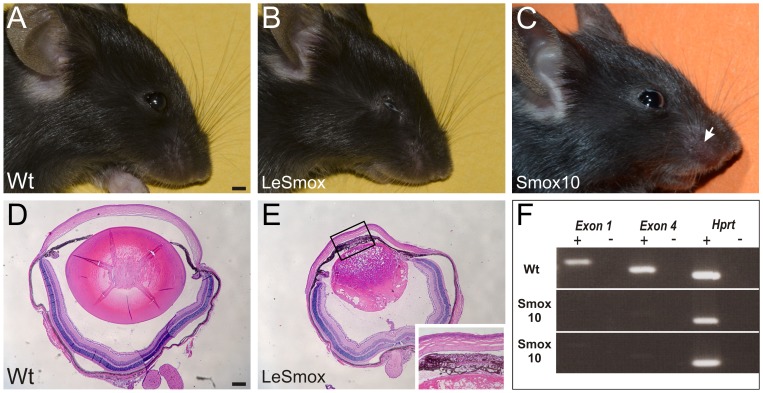
Ocular phenotype of *Smo* cKO mice. Images showing normal ocular phenotype in weanling (4 week old) wild-type (**A**) and Smox10 mice (**C**) but microphthalmia in LeSmox (**B**) mice. Smox10 mice lacked hair between whiskers (**C**, white arrow), due to ectopic Cre activity in these follicles. Histology confirmed microphthalmia in P21 LeSmox (**E**) mice compared to wild-type (**D**). The phenotype was characterized by abnormal lens development, reduction of the anterior chamber, overlapping irises and fusion of the iris to the lens (E, inset). **F.** RT-PCR showed no expression of *Smo* gene in two independent Smox10 lenses at P2, using primers specific for exons1 or 4, but normal expression of *Hprt*. Presence (+) or absence (−) of reverse transcriptase in reactions is indicated above the lanes. Scale bar **A–C**, 2 mm; **D–E**, 50 µm; inset, 90 µm.

To determine if Hh signalling plays a role in lens development we deleted *Smo* at two different embryonic stages using the LeCre and MLR10 Cre lines. Mice harbouring the LeCre transgene and both floxed *Smo* alleles (LeSmox) showed a distinct microphthalmia phenotype (Fig. 3B, E), whereas Smox10 mice, harbouring the MLR10 Cre and floxed *Smo* alleles appeared to have normal eyes (Fig. 3C). Smox10 mice did exhibit loss of hair between the whiskers, consistent with this Cre transgene being ectopically expressed in nasal hair follicles [39], [40] and previous studies indicating a requirement for *Smo* and Hh signalling in skin stem cells [44]–[46]. To confirm that *Smo* had been deleted in lenses of Smox10 mice we carried out RT-PCR experiments with primers directed to both exon 1 and exon4 and found no expression of *Smo* mRNA in Smox10 neonatal lenses (Fig. 3F). Similarly, *in situ* hybridisation using the exon 1 *Smo* probe showed specific loss of RNA from Smox10 lenses at E13.5, but not in other ocular tissues (Fig. S1). Further analyses of embryonic Smox10 mice at E14.5 showed no changes in gross morphology by H&E staining or in the expression of epithelial (E-cadherin), fibre (β-crystallin) or proliferation (phospho-histone 3) markers (Fig. S2).

Histological examination of P21 LeSmox mouse eyes showed overlapping irises and gross abnormalities of the lens. The lenses of these mice appeared to lack an anterior epithelium and the fibre mass was abnormal with disorganized and poorly elongated fibre cells as well as evidence of fibre cell degeneration (Fig. 3E). In many areas, the iris was adherent to the anterior lens and the cornea, leading to a poorly formed anterior chamber (inset, Fig. 3E). To confirm loss of *Smo* expression in LeSmox lenses we carried out *in situ* hybridsation at E11.5 and E12.5 (Fig. S3). In Wt eyes *Smo* expression was detected in the early anterior lens vesicle at E11.5 and also in the optic cup (Fig. S3A). Decreased staining was evident in the LeSmox lens vesicle but not optic cup at E11.5 (Fig. S3B). Consistent with this, RT-PCR of dissected lens vesicles at E13.5 showed distinct *Smo* expression in the Wt but not the LeSmox mutant (Fig. S3C).

**Figure 4:**
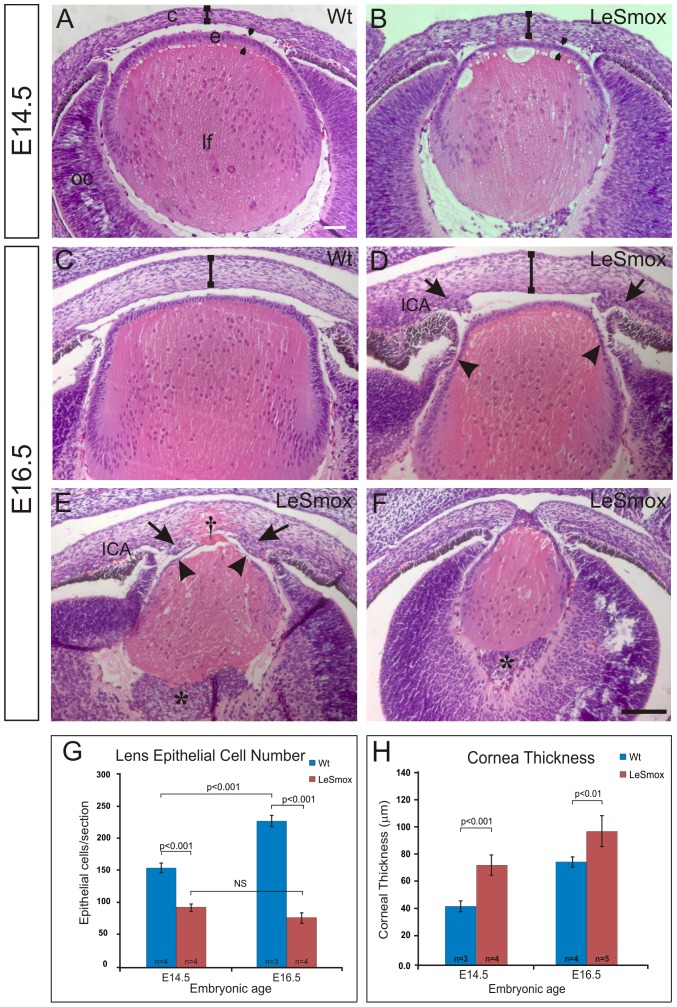
Embryonic ocular phenotype of LeSmox mutant mice. Histology of wild-type (**A, C**) and LeSmox (**B, D, E, F**) eyes at E14.5 (**A, B**) and E16.5 (**C–F**), showing reduction of epithelial thickness and size of lens at E14.5 and almost complete loss of the lens epithelium (arrowheads) by E16.5 (D,E). The thickness of the cornea is indicated by ‘I’. In some cases (**E**) the anterior lens ruptured, resulting in extrusion of lens cells into the cornea (†), whereas on other rare occasions there was a Peter's anomaly (**F**). In almost all LeSmox eyes at E16.5 (**E, F**), aberrant cells (*) were seen to accumulate near the posterior pole of the lens, in the vitreal chamber, and also in the mesenchyme (arrows) near the irido-corneal angle (ICA) **G,H.** Quantification of lens epithelial cell number (**G**) and thickness of the cornea (**H**) in LeSmox eyes compared to Wt. Abbreviations: c, cornea; e, epithelium; OC, optic cup; ICA, iridial-corneal angle. Scale bar, **A–F**, 100 µm.

Histological examination of embryonic stages indicated a variable phenotype, with occasional embryos showing a more severe phenotype than others (Fig. 4). This was particularly evident at E16.5, where some embryos showed rupture of the lens anteriorly (Fig. 4E) or, in rare cases, a Peter's anomaly with fusion of the lens and cornea via a central stalk (Fig. 4F). In this study, we did not analyse embryos that showed a Peter's anomaly or rupture of the lens into the anterior cornea. The first detectable structural phenotype was evident at E14.5 where the lens was reduced in size and had a thinned epithelium (Fig. 4A, B). The cornea at this stage appeared thicker than the Wt (Fig. 4A, B). Similarly at E16.5, the lens was noticeably smaller than the Wt lenses with a thinned epithelium, particularly in the equatorial regions (Fig. 4D). In lenses with more severe phenotype, the epithelium was grossly thinned and fibre differentiation appeared abnormal with disorganization of the fibre mass and evidence of rupture of the anterior lens into the corneal stroma or a Peter's anomaly (Fig. 4E, F).

The LeSmox cornea at E16.5 (Fig. 4D) was also considerably thicker than Wt (Fig. 4C) and in the more severe phenotypes the cornea was grossly thickened due to apparent rupture of the lens into the anterior cornea (Fig. 4E). In the LeSmox mutants there was evidence of abnormal accumulations of mesenchymal cells in the corneal-iridial angle (ICA) and within the corneal stroma (Fig. 4DE) as well as in the vitreous (Fig. 4E, F).

Quantification of epithelial cells in the lens epithelium of E14.5 and E16.5 lenses showed a significant decrease in cell number (Fig. 4G), consistent with the apparent thinned LeSmox lens epithelium (Fig. 4B, D–F). Similarly, measurement of corneal thickness showed that the corneas of LeSmox eyes were significantly thicker (Fig. 4H) at both E14.5 and E16.5. To ensure this phenotype was not associated independently with the presence of the LeCre transgene, we examined E16.5 mice that were heterozygous for both the LeCre transgene and the floxed *Smo* allele (*Smo^Wt/fl^/LeCre^+/−^*). Double heterozygous mice showed no significant difference (p>0.05) in corneal thickness (74.7±7.2 µm; n = 6), compared to Cre^−^, wild-type mice (75.3±3.5 µm; n = 4; Fig. 4H). Similarly, there was no significant difference in (p>0.05) in thickness of the anterior lens epithelium between double heterozygous mice (15.6±0.8 µm; n = 6), and control, Cre^−^ wild-type mice (15.8±0.2 µm; n = 3). By contrast, the lens epithelial thickness in LeSmox mutant mice (9.5±0.8 µm; n = 7) was significantly reduced (p = 0.0002), consistent with the reduced numbers of epithelial cells in these lenses (Fig. 4G).

### Patterns of fibre differentiation are not grossly disrupted in LeSmox mice

**Figure 5:**
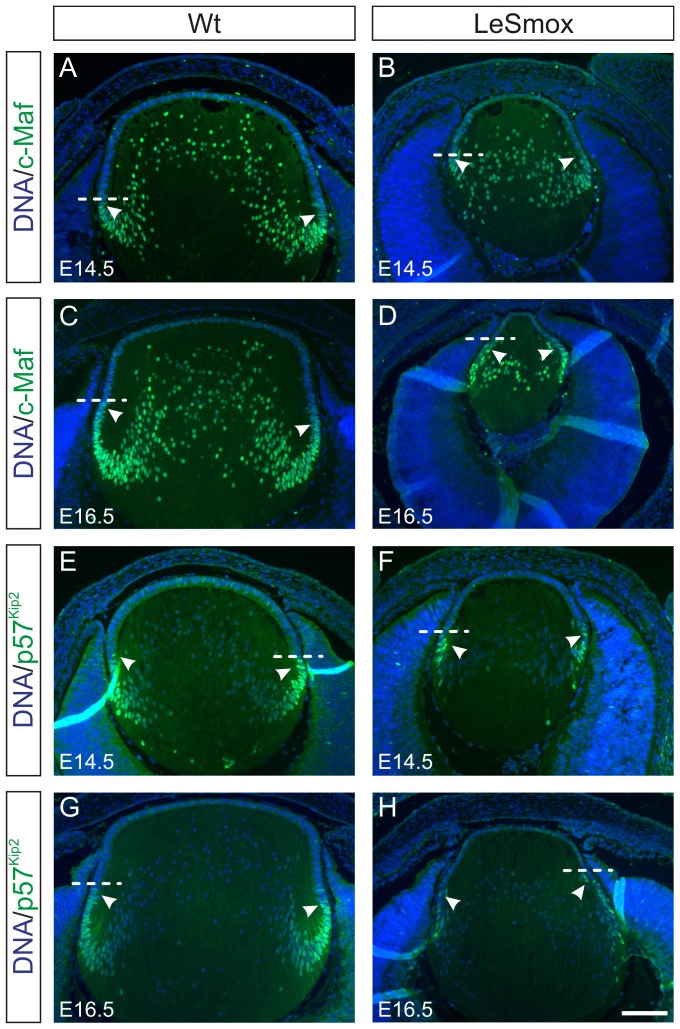
Expression of fibre cell markers in LeSmox lenses. Expression of c-Maf (**A–D**) and p57^Kip2^ (**E–H**) proteins in Wt (**A, C, E, G**) and LeSmox (**B, D, F**, **H**) lenses at E14.5 (**A, B, E, F**) and at E16.5 (**C, D, G, H**). Reactivity for both c-Maf and p57^Kip2^ proteins were detected in the nuclei of early differentiating fibre cells (arrowheads) below the lens equator (dashed line) in Wt and in LeSmox lenses in similar positions. Scale bar, **A–H** 100 µm.

As fibre differentiation appeared to be compromised in LeSmox mice we examined the expression of two key fibre cell markers, c-Maf, which is a transcription factor required for crystallin expression and p57^Kip2^, a cyclin-dependent kinase inhibitor, required for cell cycle exit and fibre differentiation [47]. Both proteins are normally expressed in the fibre cell nuclei just below the lens equator at E14.5 (Fig. 5A, E) and E16.5 (Fig. 5C, G) in wild-type lenses. While the numbers of positively stained cells appeared reduced in LeSmox lenses, the pattern of expression was not markedly different from that seen in the wild-type; distinctly labelled nuclei were present in the transitional zone below the lens equator (Fig. 5B, D, F, H) at both ages.

### Selective decrease of FoxE3, Ptch1 and Hes1, but not Pax6 or E-cadherin in LeSmox lenses

**Figure 6:**
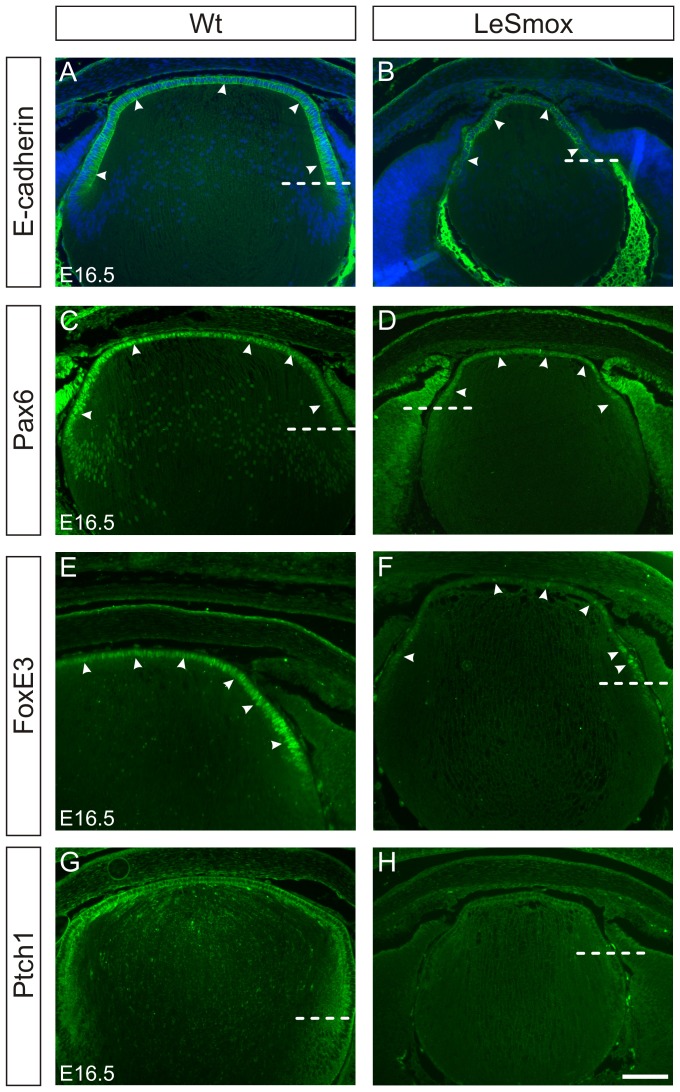
Expression of epithelial cell markers in LeSmox lenses. Expression of E-cadherin (**A, B**), Pax6 (**C–D**), FoxE3 (**E–F**) and Ptch1 (**G, H**) proteins in Wt (**A, C, E, G**) and LeSmox (**B, D, F, H**) lenses at E16.5. **A–D.** Reactivity for both E-cadherin and Pax6 proteins is detected in epithelial cells (arrowheads) anterior to the lens equator (dashed line) in Wt and in LeSmox lenses. While the extent of the epithelium in LeSmox mice is decreased, the patterns of protein expression are similar to Wt. **E–F.** FoxE3 is normally detected as a nuclear reactivity in wild-type lens epithelium (arrowheads) but in LeSmox lenses the reactivity is reduced and is absent from many nuclei. The strongest FoxE3 reactivity is detected in equatorial nuclei. **G–H.** Ptch1 is predominantly detected in Wt epithelial cells and declines as cells commenced differentiation at the equator (**G**). In LeSmox lenses, reactivity for Ptch1 is greatly reduced and virtually absent, indicating reduced Hh signalling (**H**). Dashed line indicates position of the lens equator in each image. Scale bar, **A–H** 100 µm.

Examination of the epithelial markers E-cadherin and Pax6 showed no major changes in the patterns of protein expression. Membrane-associated reactivity for E-cadherin is normally restricted to the epithelial cells anterior to the equator in E16.5 (Fig. 6A). As shown previously, nuclear reactivity for Pax6 is most strongly detected in the anterior epithelium of wild-type lenses and declines in the differentiated fibre cells (Fig. 6C). In LeSmox lenses at E16.5, the epithelium was depleted, resulting in slightly less intense staining; however the patterns of E-cadherin and Pax6 expression were similar to the wild-type (Fig. 6B, D). Similar results were seen in E14.5 embryos (not shown). As it has previously been shown that activation of *Smo* in the lens could induce ectopic activation of FoxE3 expression [35], we also examined FoxE3 localization in LeSmox lenses. As described previously, the reactivity for FoxE3 in the Wt lens is detectable in the nuclei of lens epithelial cells, but not in fibre cells (Fig. 6E). In the LeSmox lenses at E16.5, the FoxE3 reactivity was markedly reduced or absent in many sections, the most strongly labelled nuclei were detectable at the lens equator (Fig. 6F). To confirm that loss of *Smo* affected the canonical Hh signalling pathway, we examined the expression of Ptch1, which is a direct target of the Hh pathway, in LeSmox lenses. Similar to results presented in Figure 2, Ptch1 reactivity was strongly detectable in the epithelium and early fibres of Wt lenses (Fig. 6G). However, in the LeSmox lenses, the reactivity for Ptch1 was markedly reduced or absent (Fig. 6H) and is consistent with the loss of *Smo* and inhibition of Hh signalling.

**Figure 7:**
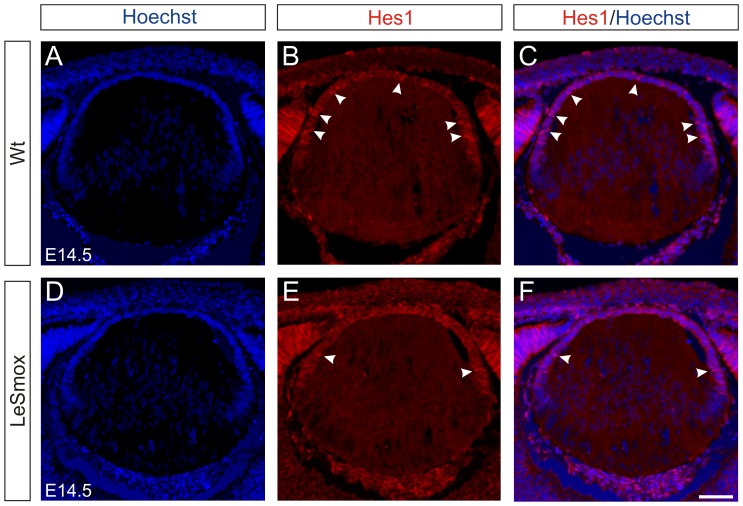
Expression of Hes 1 in LeSmox lenses. Frozen sections stained with Hoechst dye (**A, D**), Hes1 antibody (**B, E**) and respective merged images (**C, F**) from Wt (**A–C**) and LeSmox (**D–F**) lenses at E16.5. **A–C**. In the wild-type lenses, nuclear reactivity for Hes1 was detected strongly in epithelial nuclei (arrowheds). **D–F.** Reactivity for Hes1 was mainly cytoplasmic in the LeSmox lenses with only occasional nuclei at the equator showing intense Hes1 reactivity. Scale bar. 100 µm.

Another marker for epithelial cells is the Hes1 protein, which is known to be downstream of Notch signalling in lens [48], but also downstream of Hh signals in the retina, independently of Notch signaling [49]. In Wt lenses, Hes1 localizes to the epithelial layer and is seen as both cytoplasmic and nuclear reactivity (Fig. 7A–C). However, in the LeSmox lenses, mainly cytoplasmic staining is present in the epithelium with only occasional nuclei near the equator showing nuclear reactivity (Fig. 7D–E).

### Altered patterns of lens epithelial proliferation in Le Smox mice

**Figure 8:**
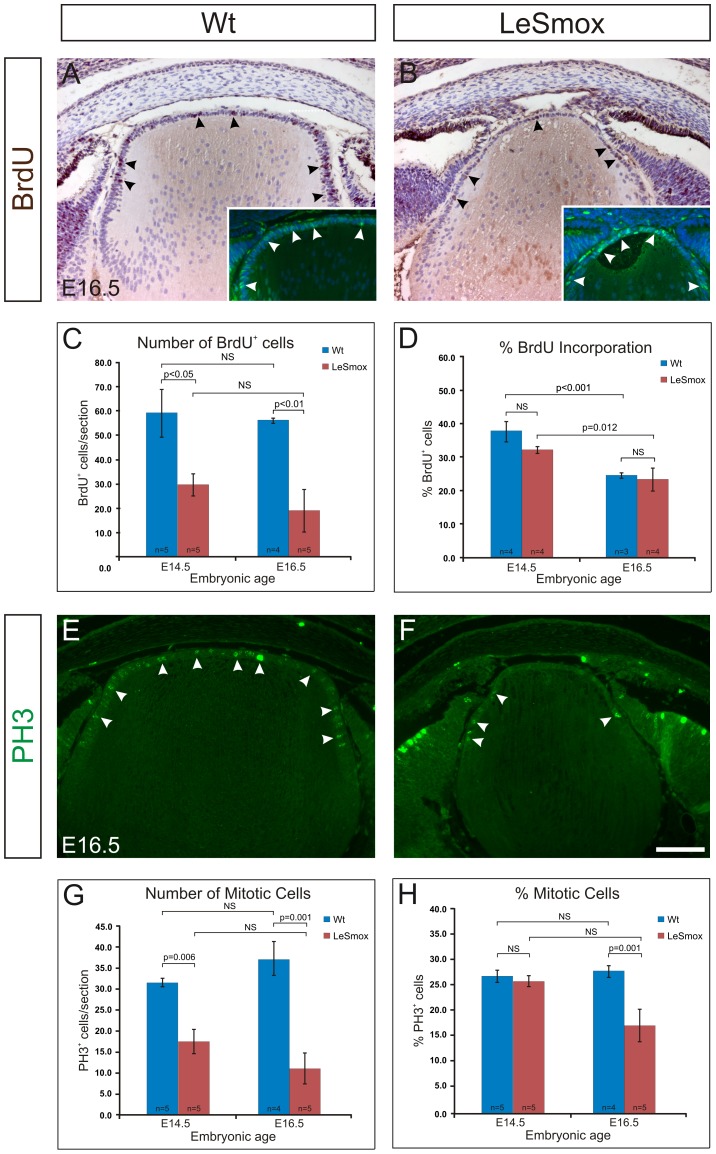
Epithelial cell proliferation in LeSmox lenses. BrdU labelling in wild-type (**A**) and LeSmox (**B**) lenses reveals numerous BrdU^+^ nuclei in the epithelial layer (black arrowheads). Consistent with this, numerous cyclin D1+ nuclei are detected (insets A, B; white arrowheads). Immunolabelling for phospho-histone-3 (PH3) reveals numerous PH3^+^ nuclei in the wild-type (**E**) but reduced labelling in the LeSmox (**F**) lenses. Quantification of BrdU^+^ (**C**) and PH3^+^ (**G**) nuclei revealed significant changes in the total numbers of positive cells in each section. However, quantification of the percentage of cells labelled (**D, H**) indicated no significant changes in BrdU incorporation (**D**) but significantly reduced PH3 labelling index in LeSmox lenses at E16.5 compared to Wt (**H**). Scale bar, **A, B, E, F, insets**, 100 µm.

The depleted epithelium seen at E14.5 and E16.5 in LeSmox lenses suggested a defect in cell proliferation or increased cell death. To examine epithelial proliferation we used BrdU incorporation to label S phase and immunohistochemistry for cyclin D1 and phosphohistone-H3 to label cells undergoing G1/S phase transition or M phase, respectively (Fig. 8). In both Wt and LeSmox lenses, BrdU labelling was restricted to the epithelium, with fewer cells appearing to be labelled in the LeSmox mutants at E16.5 (Fig. 8B). Surprisingly, while the total number of BrdU^+^ cells in the epithelium was significantly decreased at both E14.5 and E16.5 in the LeSmox lenses (Fig. 8C), no major changes were observed in the percentage of BrdU labelled cells in LeSmox lenses compared to wild-type (Fig. 8D). The reduced number of BrdU^+^ cells appears to be matched by the reduction in the overall numbers of epithelial cells in the LeSmox mutant epithelium (see Fig. 4G). This suggested that the rate of cells entering S phase did not change in the LeSmox mutants. Consistent with this we detected similar levels of cyclin D1 staining in LeSmox lenses and Wt lenses (insets, Figs 8A, B). To determine whether there might be changes in progression of cells through M-phase of the cell cycle, we examined the pattern of phosphohistone-H3 (PH3) reactivity (Figs. 8E, F). As demonstrated previously, the pattern of PH3 reactivity in wild-type lenses is restricted to the anterior epithelium (Fig. 8E). In LeSmox lenses there was a marked and significant reduction in the number of PH3^+^ cells per section at both E14.5 and E16.5 (Fig. 8G). This was particularly so at E16.5, with very few PH3^+^ nuclei detected. (Fig. 8F, G). Quantification confirmed a significant decrease in the percentage of PH3^+^ nuclei labelling in LeSmox compared to Wt lenses at E16.5 but not at E14.5 (Fig. 8H). These data suggest that loss of *Smo* in the LeSmox lens results in a reduction in the total number of epithelial cells in the epithelium and a reduced ability of epithelial cells to progress through the G2/M phase transition, particularly at E16.5.

### Increased epithelial cell death in LeSmox mice

**Figure 9:**
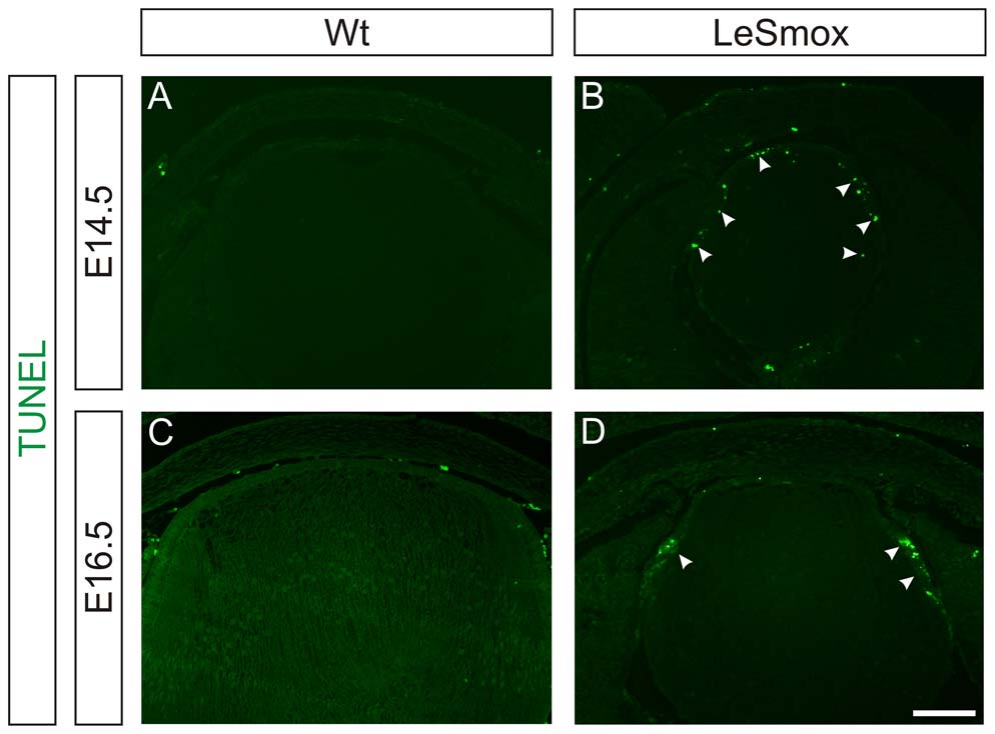
Cell death in LeSmox lenses. TUNEL reaction in wild-type (**A, C**) and LeSmox (**B, D**) lenses at E14.5 (**A, B**) and E16.5 (**C, D**). In wild-type lenses (**A, C**) no distinct TUNEL^+^ nuclei were detected. In E14.5 LeSmox lenses (**B**) numerous TUNEL^+^ nuclei were detected throughout the epithelium and occasionally in fibre cells in the transitional zone. At E16.5 (**D**), TUNEL^+^ nuclei were concentrated in the equatorial zone at or just anterior to the equator. Scale bar, **A–D**, 100 µm.

As the epithelial deficiency in the LeSmox lenses at E14.5 could not be completely explained by altered cell cycle progression at G1/S and G2/M, we also examined whether the loss of epithelial cells was due to cell death, using the TUNEL assay. As described previously, TUNEL^+^ cells are not detected in wild-type lenses at E14.5 or at E16.5 (Fig. 9A, C). However, in LeSmox lenses numerous TUNEL^+^ nuclei were detected in the anterior epithelium at E14.5 and at E16.5 (Fig. 9C, D). While at E14.5 the TUNEL^+^ cells were distributed throughout the epithelium, they were concentrated at the lens equator at E16.5 (Fig. 9D), suggesting there is a central to peripheral progression of cell death in the lens epithelium. In the cornea, very few TUNEL^+^ cells are seen at either E14.5 or E16.5. However in the LeSmox eyes, particularly at E14.5, TUNEL^+^ cells are present in the corneal epithelium, stroma and the aberrant cells that line the anterior chamber (Fig. 9B). Occasional TUNEL^+^ cells are also present in the corneal epithelium at E16.5 (Fig. 9D).

### Delayed corneal endothelial differentiation in LeSmox mice

**Figure 10:**
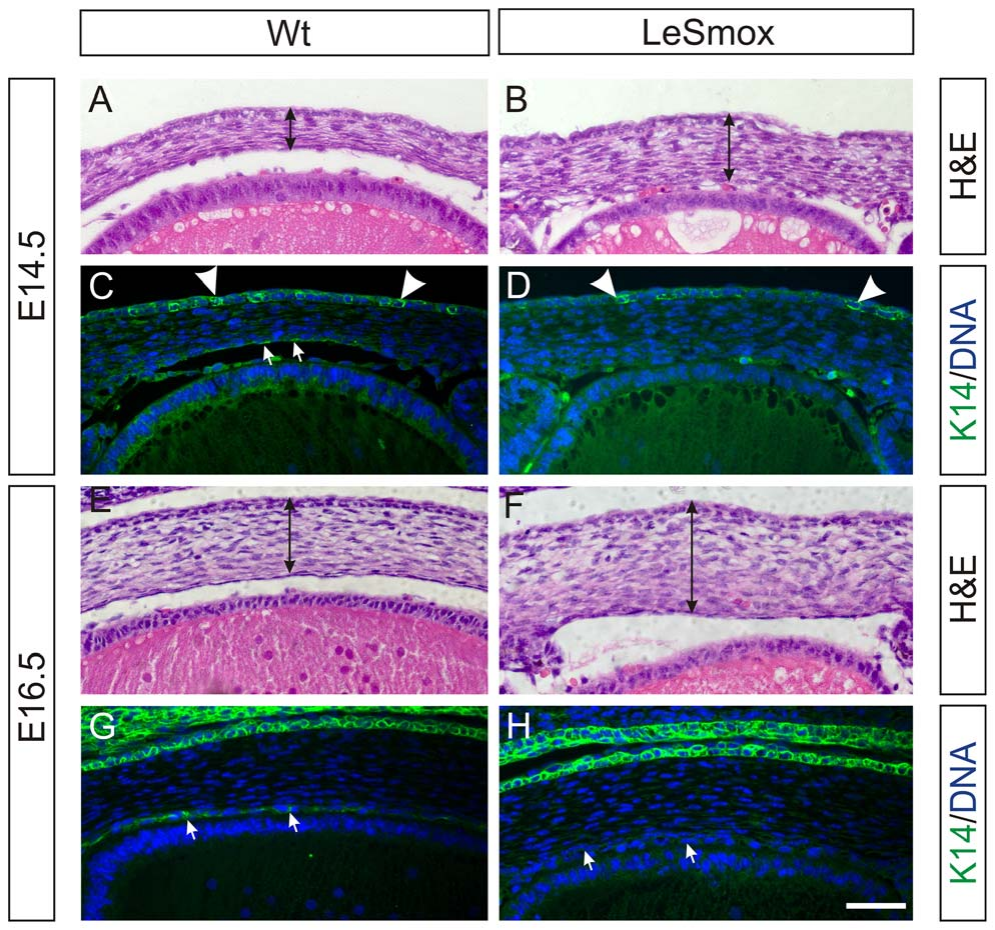
Altered cornea development in LeSmox eyes. Haematoxylin & eosin staining (**A, B, E, F**) and keratin-14 immunofluorescence (**C, D, G, H**) in wild-type (**A, C, E, G**) and LeSmox (**B, D, F, H**) anterior segment at E14.5 (**A–D**) and E16.5 (**E–H**). The LeSmox cornea is noticeably thicker (double arrows) than the Wt cornea at E14.5 (**A, B**) and at E16.5 (**E, F**), due to additional stromal layers, and lacks a defined endothelial layer. **C.** In the Wt cornea at E14.5, keratin-14 labelling is clearly detectable in the epithelial layer (arrowheads) but only weakly detectable in the endothelium (small arrows). **D.** In the LeSmox cornea at E14.5, the distinct keratin-14 labelling is present in the epithelium (arrowheads) but absent in the endothelium. Similarly, at E16.5 (**G, H**), distinct keratin-14 labelling is present in the epithelium of Wt (**G**) and LeSmox (**H**) epithelium and Wt endothelium (**G**, small arrows) but is completely absent from the LeSmox endothelium (**H**, small arrows). Scale bar, A–H, 50 µm.

The histological analyses suggested that the development of the cornea, particularly the endothelial and stromal layers, was disrupted in LeSmox eyes. Higher magnification microscopy of the mutant corneas shown in Figure 4 confirms the increased thickness of the stromal layer at both E14.5 and E16.5 (Fig. 10B, F). The corneal epithelium appears similar in both the Wt and the LeSmox mutant. However, while squamous endothelial cells are visible in the Wt at E14.5 (Fig. 10A), these appear to be absent in the LeSmox mutant (Fig. 10B). At E16.5, the LeSmox corneas are noticeably thicker than the Wt, due to increased stromal layers (Fig, 10E, F), and the corneal epithelium and endothelium are present in both Wt and LeSmox corneas.

To investigate corneal differentiation, we examined the expression of keratin-14 (K14) and keratin-12 (K12), which are markers for epithelial differentiation [50]. Consistent with earlier studies [51] we detected patchy K14 reactivity in the E14.5 corneal epithelium (Fig. 10C), which was more intense and more extensive at E16.5 (Fig. 10G). Weak reactivity was also present in the developing endothelium at both ages. In the LeSmox corneas, the pattern of reactivity in the corneal epithelium was similar to Wt but the endothelial staining pattern was absent at both E14.5 and E16.5. Examination of keratin-12 reactivity in the epithelium Wt and LeSmox corneas showed no difference (not shown), with distinct labelling of the corneal epithelial cells at both ages, suggesting corneal epithelial differentiation was normal.

### Aberrant migration of mesenchymal cells into the LeSmox cornea

In addition to the extra layers of stromal cells in the cornea, a noticeable feature of LeSmox mutant eyes was the aberrant groups of cells located in the vitreous chamber near the posterior pole of the lens (Fig. 4E, F). Previous studies have shown that neural crest (NC) cells populate the periocular regions, including the matrix adjacent to the overlying surface ectoderm to form the corneal stroma and endothelium [7]. Some mesodermal cells also migrate into the eye and contribute to endothelial cell populations of the hyaloid vasculature and pericytes [52]. However, large cell accumulations in the vitreous do not occur unless there is a small or absent lens [4], [7]. To investigate whether the aberrant cells in the cornea and vitreous were of NC origin, we examined the expression of Nrp2, which is a known marker for cranial NC cells that migrate to the eye [3], [53].

**Figure 11:**
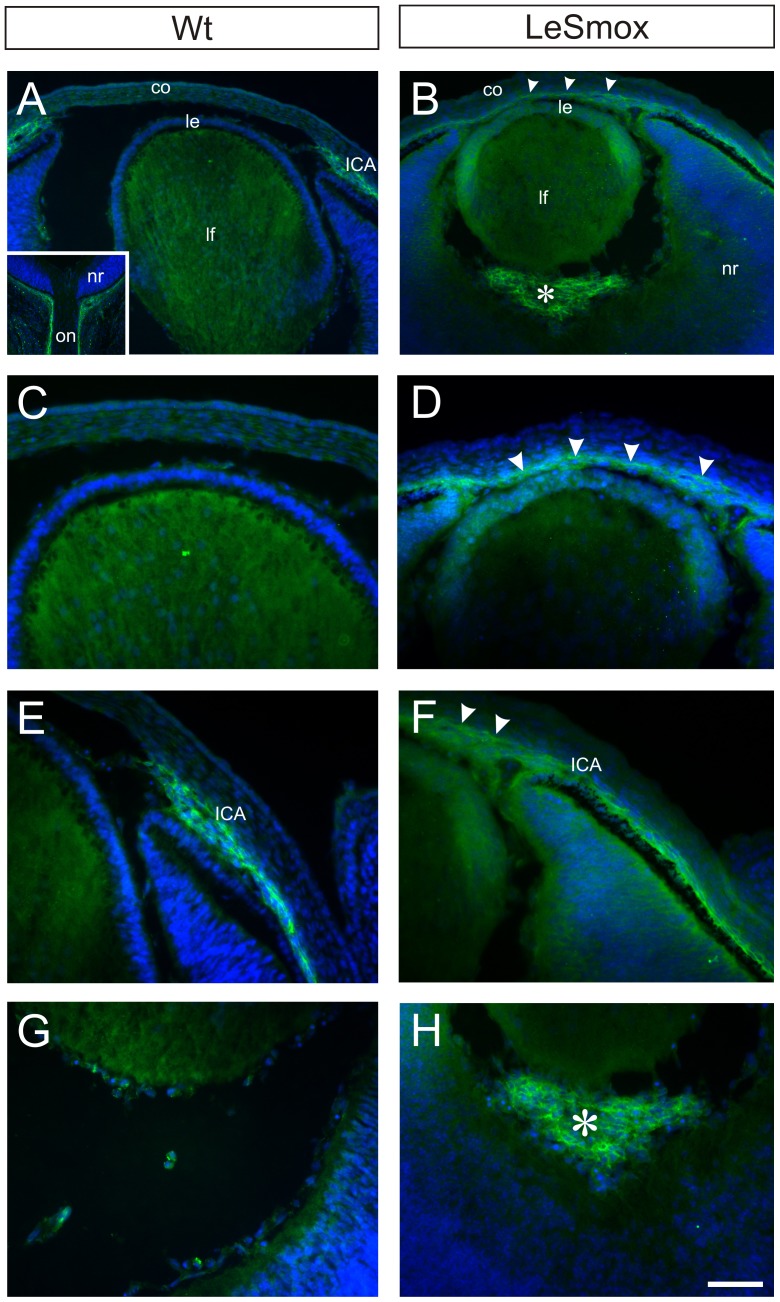
Abnormal migration of Nrp2^+^ cells in LeSmox eyes. Nrp2 immunofluorescence in frozen sections from Wt (**A, C, E, G**) and LeSmox (**B, D, F, H**) eyes at E14.5. In Wt eyes, Nrp2^+^ mesenchymal cells are found in the iridial-corneal angle (**A**, **E;** ICA) and lining the external surface of the optic cup and optic nerve (inset, **A**), but not in the cornea (**C**) or rarely in the vitreous (**G**). The Wt lens fibres are weakly reactive (**A, C, E**). By contrast, in Le Smox eyes, Nrp2^+^ mesenchymal cells extend abnormally from the ICA across the inferior layers of the cornea (arrowheads, **B**, **D**, **F**) and are abundant in abnormal cell clusters (*) in the vitreous (**B**, **H**). Abbreviations, co, cornea; ICA, iridial-corneal angle; le lens epithelium; lf, lens fibres; nr, neural retina; on, optic nerve. Scale bar, **A–B**, 100 µm; **C–H**, 50 µm; inset A, 250 µm.

In frozen sections from Wt eyes at E14.5, strong reactivity for Nrp2 was detected in mesenchymal cells of the iridial-corneal angle (ICA). These cells were restricted to the ICA and to mesenchyme covering external optic cup and optic nerve (Fig. 11A, E), but were never seen in the anterior chamber medial to the iridial margin (Fig. 11C, E). Weak reactivity was also detected in the lens fibre mass, but not in the epithelium, of Wt lens (Fig. 11A) and only occasional Nrp2^+^ cells were found in the vitreous (Fig. 11 G). However in the LeSmox eyes, Nrp2^+^ cells were found to extend into the anterior chamber, along the inferior surface of the cornea where the endothelium normally develops (Fig. 11B, D) and in the abnormal cell mass in the vitreous (Fig. 11H). The epithelium of the mutant lenses also showed weak reactivity for Nrp2. These findings suggest that in the LeSmox mutants there is abnormal migration of Nrp2^+^ NC cells into the developing cornea and vitreous.

## Discussion

Previous studies have shown that a central source of Shh in anterior mesoderm is required for separation of the eye fields during the early events that specify ocular precursors following gastrulation [14]–[17], [54]. Subsequent studies have also shown that Hh signals are required to pattern the dorso-ventral axis of the optic cup [19], [20]. In this study we show that Hh signalling is required during a discrete, early stage of lens differentiation, following eye induction and early ocular morphogenesis.

### Discrete timing of *Smoothened* requirement during lens differentiation

Together with studies by Kerr et al. [35] and Bakrania et al. [30] this study provides evidence that the Hh pathway is expressed and plays a role during early stages of mammalian lens differentiation. The key receptor (*Ptch1*), transmembrane signalling protein (*Smo*) and transcription factors (*Gli2, Gli3*) are all present in the embryonic, differentiating lens and their expression patterns are consistent with a functional role soon after the lens vesicle has formed. However, the period during which the Hh pathway is active appears to be restricted. Expression of Gli1 and Gli2 was absent postnatally and their patterns of expression between E12.5 and E13.5 suggest changes in Hh signalling activity.

While the antibodies used cannot distinguish the activator or repressor forms of these transcription factors, the presence of nuclear Gli2 from E12.5 suggests the pathway may be active at these stages, particularly as Gli2 is known to be an activator of the pathway. By contrast, as Gli3 functions predominantly as a repressor, the accumulation of Gli3 in the nuclei of lens cells after E13.5 suggests that the pathway is progressively inactivated through these stages. Further work needs to be done to identify the activity and role of these Gli proteins in the developing lens.

Consistent with the localisation data, the lack of a phenotype in the Smox10 mouse indicates that *Smo* is not required after ∼E13.5, when the MLR10 Cre transgene is known to efficiently delete genes from the lens [38]–[40]. While the lack of a suitable antibody prevented us from documenting exactly when the Smoothened protein is lost from these lenses, the *in situ* hybridisation data indicated there was complete loss of *Smo* mRNA expression by E13.5 and RT-PCR experiments confirmed that *Smo* was not expressed neonatally in the mutant Smox10 lenses.

By contrast, deletion of *Smo* using the LeCre transgene, which is active from E9.5, resulted in a distinct lens phenotype. Combined with the Smox10 data, this suggests that *Smo* is required between E9.5 and E13.5. Our *in situ* hybridisation data further refines this period, as *Smo* mRNA expression is reduced at E11.5 and completely absent by E13.5, suggesting a requirement for *Smo* signalling from ∼E12. Importantly, the expression of the ligand, *Shh*, in the optic cup has been reported to be initiated at about this stage [55]. Similarly, the studies by Kerr et al., demonstrated that conditional activation of the Smoothened protein, from E8.5 in the murine pre-lens ectoderm results in abnormal epithelial cell proliferation, dysregulation of the cell cycle, disrupted fibre differentiation and abnormal expression patterns for FoxE3, Pax6 and c-Maf only from E12.5 onwards [35].

As Ptch1 and Smo are present in lens at all embryonic and postnatal stages studied, the activity of Hh signalling is unlikely to be mediated by changes in expression of these transmembrane proteins. Intriguingly, mice that lack the co-receptor *Cdon* have very similar lens phenotype, with markedly reduced cell proliferation and increased epithelial cell death. As the expression of this gene in the lens appears to be restricted to the lens placode and early lens vesicle stages (E10.5–E12.5) [56] and is known to enhance Shh signaling [57], it is plausible that the transient expression of this co-receptor at these stages underlies the transient activity of the Hh pathway in the lens, documented here.

Intriguingly, our analyses show that the effects of *Smo* deletion in the LeSmox mice do not manifest in the lens until ∼E14.5, which is approximately 5 days after recombination occurs with LeCre and approximately 2–3 days after the documented loss of *Smo* mRNA, suggesting that the effects of Hh signalling in the lens take some time to materialise. At this stage it is unclear whether this is an indirect effect on another signalling pathway, downstream transcription factors (e.g. FoxE3, see below) or whether the Smoothened protein is robust and persists in the lens after the gene is deleted. In the retina, Hh-Gli2 signalling has been shown to regulate the transcription factor, *Hes1* [49], *w*hich is also central to the Notch pathway. In the lens, the Notch pathway, acting via Hes1, is an important regulator of epithelial cell proliferation [48]. Our finding of reduced nuclear Hes1 reactivity in the LeSmox lenses raises the interesting possibility that the effects of the Hh pathway may be via interaction with the Notch pathway and this is reinforced by the fact that the phenotype of the LeSmox lenses bears some similarity to Notch pathway mutants (*Notch1*, *Rbpj*) with respect to the altered proliferation of epithelial cells [58].

### Loss of *Smo* affects G2-M phase transition

The BrdU incorporation analyses indicated that there were no major changes in S phase entry in LeSmox mutants. However, reduced PH3 labelling in these mutants indicated that the transition from G2/M was compromised. Consistent with this, we also saw fewer fibre cells labelled with p57^Kip2^ and c-Maf, suggesting that fewer fibre cells were being generated in these smaller lenses. However, the pattern of expression of these proteins did not seem to be markedly altered at E14.5 or E16.5, indicating that the spatial pattern of fibre differentiation was still relatively normal, albeit slowed.

Many studies have examined the role of Shh signalling in control of cell cycle and most indicate that Hh signals activate the G1/S phase transition [59], [60]. Similarly in the lens, activation of Hh signals causes an increase in cyclins D1/D2, which activate the G1/S phase transition [35]. However, the results of the current study suggest that loss of Hh signals affect G2/M and not G1/S phase transition in lens epithelial cells. One reason that loss of *Smo* may not have affected G1/S transition is that various growth factors in the eye, such as FGFs, PDGF, IGF and Wnts, can regulate this cell cycle check-point [1], [8] and may thus compensate for any cell cycle dysregulation by loss of Hh signals. While activation of Hh signalling can affect accumulation of cyclin B1 [61], which together with Cdk1 (cdc2) constitutes the mitosis promoting factor (MPF), the mechanism by which Hh signalling regulates G2/M transition is less well understood. In cell lines, it has been shown that the Shh pathways can activate the G2/M transition by inhibiting the interaction of Ptch-1 with cyclin B1 [62]. In the absence of Shh, Ptch-1 binds cyclin B1, retaining it in the cytosol and preventing its interaction with nuclear Cdk1. However, addition of Shh disrupts this interaction with Ptch1 and permits nuclear translocation of cyclin B1 and formation of the active MPF. An alternative mechanism by which Shh may regulate G2/M transition is via expression of CDC25 proteins, a family of phosphatases that regulate the activity of Cdk1/cyclin B complexes. In chick embryos it was shown that modulation of Hh signalling can regulate the expression of CDC25B in neural tube and developing limb bud [63]. Further studies will need to be undertaken to determine whether CDC25B is expressed in lens and is regulated by manipulations of Hh signalling in both LeSmox lenses and lenses with activated *Smo* [35].

### A role for *Smo* in regulating lens gene expression

Kerr et al. showed that constitutive activation of the Hh pathway by conditional mutation of *Smo* resulted in promotion of epithelial phenotype and failure of epithelial cells to differentiate [35]. In particular, they showed there was ectopic induction of FoxE3 and Pax6 expression in the fibre compartment of the lens. However in this study, the expression patterns of two epithelial markers, Pax6 and E-cadherin, appeared relatively normal, suggesting that Hh signalling is not essential or required for their expression.

By contrast, expression of FoxE3 and Ptch1 were decreased in the LeSmox epithelium at E16.5. The loss of Ptch1 reactivity would be expected if Hh signalling was normally active in lens, as *Ptch1* has been shown to be a direct target of the pathway. This loss of Ptch1 reactivity is concordant with reduced Hh signalling due to *Smo* deletion in the LeSmox lenses. Similarly, the decrease in FoxE3 expression is consistent with findings by Kerr et al., (2012) and indicates that Hh signalling is not only sufficient to induce *FoxE3* but is also required for its optimal expression. That there is still some FoxE3 reactivity present in these lenses suggests there may be other factors that contribute to FoxE3 expression or that the Hh pathway is not completely inhibited in these mutant lenses or that FoxE3 is a resilient, long-lived protein. Studies of mice that lack *FoxE3* or have a loss-of-function mutation (*dyl*) reveal a phenotype that is similar to that seen in the LeSmox lenses [64], [65]. The lens epithelium in these mice fails to proliferate and undergoes apoptosis and, similar to the more severely affected LeSmox embryos, display a Peter's anomaly-like defect with a residual lens-corneal stalk. This raises the possibility that the defects seen in the LeSmox lenses are mediated by the reduced expression of FoxE3. However, it remains to be determined if Gli proteins can directly bind the FoxE3 promoter region and thus directly regulate *FoxE3* expression or whether this is an indirect effect, possibly via Notch signalling. That the Smox10 mice lack a lens phenotype suggests that at this stage there is no effect on FoxE3 expression and that there are other factors involved in regulating FoxE3 expression during later stages of lens differentiation.

The relatively normal patterns of c-Maf and p57^Kip2^ expression observed in the LeSmox mice suggest that Hh signalling does not have a primary effect on fibre differentiation. There did appear to be fewer cells stained in the transitional zones of the Le Smox lenses; however the cells that were stained appear to have the same level of staining as observed in the Wt. The most parsimonious explanation for the reduced number of c-Maf^+^ and p57^Kip2+^ cells in the smaller LeSmox lenses, is a decreased rate of fibre differentiation due to the decreased supply of epithelial cells. However, the appropriate spatial initiation of fibre-specific genes (β-crystallin, p57^Kip^, c-Maf) still occurs in these lenses.

### Disrupted Hh signalling in lens has secondary effects on corneal development

A striking feature of the phenotype in the LeSmox eyes was the increased corneal thickness. In particular at E14.5–E16.5 there were extra layers of cells in the corneal stroma, delayed formation of the endothelial layer and aberrant accumulations of cells in the vitreous. Similar phenotypes were identified in the TGFβ2-null mice [6] and in chick embryos with inhibition of Sema3A [7]. During corneal development in the mouse, both NC and mesodermal cells contribute to the formation of the corneal stroma and endothelium (see [52], [66]). Studies in chick [4], [7] have shown that the lens and more particularly, lens-derived Sema3A, functions to regulate the timing of neuropilin-expressing NC cells that migrate into the anterior eye and their subsequent differentiation into the corneal endothelium. By contrast in mice, lens-derived TGFβ2 has been implicated as a chemo-attractant in regulating NC cell migration into the cornea [6]. The results obtained here, suggest that loss of Hh signalling in the lens results in disruption of the lens-mediated effect on NC cells that express the Sema3A/Sema3F receptor, Nrp2. In the LeSmox mutants, there was aberrant migration of the Nrp2^+^ NC cells across the inferior cornea as well as into the vitreous. This suggests that loss of *Smo* has disrupted semaphorin signalling from the lens to Nrp2^+^ NC cells. However, it remains to be determined which genes are dysregulated in the LeSmox lenses that contribute to the abnormal migration of the Nrp2^+^ NC cells.

Overall, this study has shown that Hh signalling plays an important, albeit restricted, role during early murine lens development and is involved in regulating the expression of a key transcription factor (FoxE3), lens epithelial cell cycle progression and survival. Indirectly, loss of Hh signals in the lens affects the migration of neural crest cells into the cornea. These findings may have mechanistic relevance for the spectrum of ocular defects found in patients with Gorlin syndrome [31]–[34].

## Supporting Information

Figure S1 Loss of *Smo* expression in E14.5 Smox10 lenses. *In situ* hybridisation for *Smo* mRNA in Wt (**A**) and Smox10 (**B**) lenses. In Wt lenses, *Smo* mRNA is detected in lens epithelial (le) cells and early differentiating fibres (lf) below the equator (dashed line). Scale bar, 150 µm.
(TIF)

Figure S2 Smox10 lenses develop normally. Comparison of Wt (**A, C, E, G**) and Smox10 (**B, D, F, H**) lenses at E13.5, by Haematoxylin & Eosin stain (**A, B**) and immunolabelling for E-cadherin (**C, D**), β-crystallin (**E, F**) and phospho-histone 3 (**G, H**), show no abnormal changes in the Smox10 lenses. Scale bar, **A–B**, 200 µm; **C–H**, 100 µm.
(TIF)

Figure S3 Loss of *Smo* expression in LeSmox lenses. *In situ* hybridisation for *Smo* mRNA in Wt (**A**) and LeSmox (**B**) lenses at E11.5 and RT-PCR (**C**) for *Smo* and *Hprt* in isolated E13.5 lenses, showing loss of *Smo* expression in LeSmox mutants. In Wt lenses at E11.5 (**A**), *Smo* mRNA is detected in the early lens pit/vesicle, particularly in the anterior vesicle cells (arrowheads) but also in the optic cup. **B.** In the LeSmox mutants at E11.5 signal for Smo mRNA is decreased in the lens pit. **C.** In the LeSmox mutants at E13.5, no expression of *Smo* is detected in isolated E13.5 lens vesicles by RT-PCR. Plus (+) and minus (−) signs indicate presence or absence of reverse transcriptase, respectively. Scale bar: 50 µm (**A, B**).
(TIF)
