# Supplementary figures and images for: A Role for Smoothened during Murine Lens and Cornea Development
Source: PLoS One. 2014 Sep 30;9(9):e108037. doi: 10.1371/journal.pone.0108037 (PMC4182430; doi:10.1371/journal.pone.0108037)

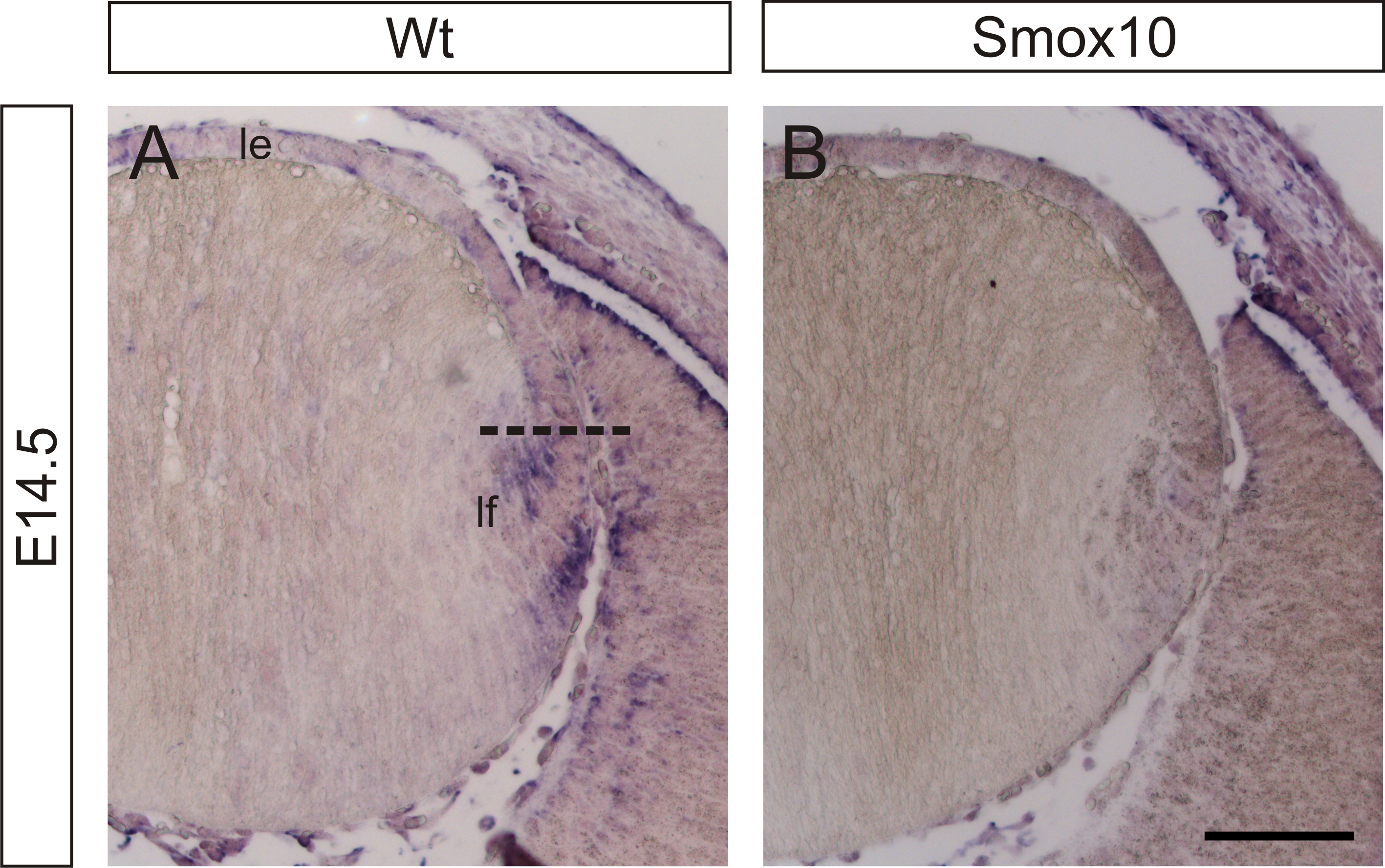

Supplement: Figure S1 — Loss of Smo expression in E14.5 Smox10 lenses. In situ hybridisation for Smo mRNA in Wt (A) and Smox10 (B) lenses. In Wt lenses, Smo mRNA is detected in lens epithelial (le) cells and early differentiating fibres (lf) below the equator (dashed line). Scale bar, 150 µm. (TIF) [file pone.0108037.s001.tif]

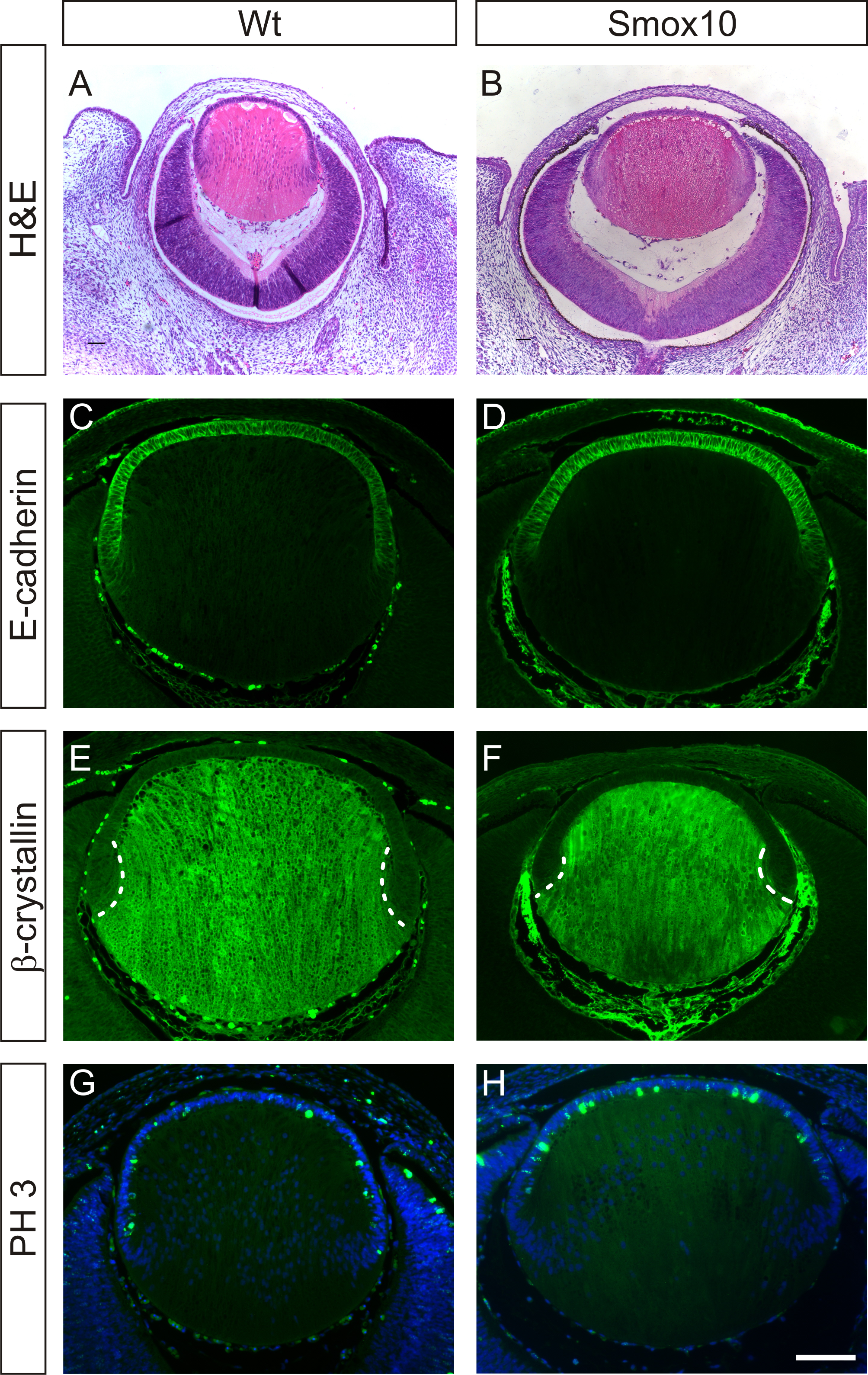

Supplement: Figure S2 — Smox10 lenses develop normally. Comparison of Wt (A, C, E, G) and Smox10 (B, D, F, H) lenses at E13.5, by Haematoxylin & Eosin stain (A, B) and immunolabelling for E-cadherin (C, D), β-crystallin (E, F) and phospho-histone 3 (G, H), show no abnormal changes in the Smox10 lenses. Scale bar, A–B, 200 µm; C–H, 100 µm. (TIF) [file pone.0108037.s002.tif]

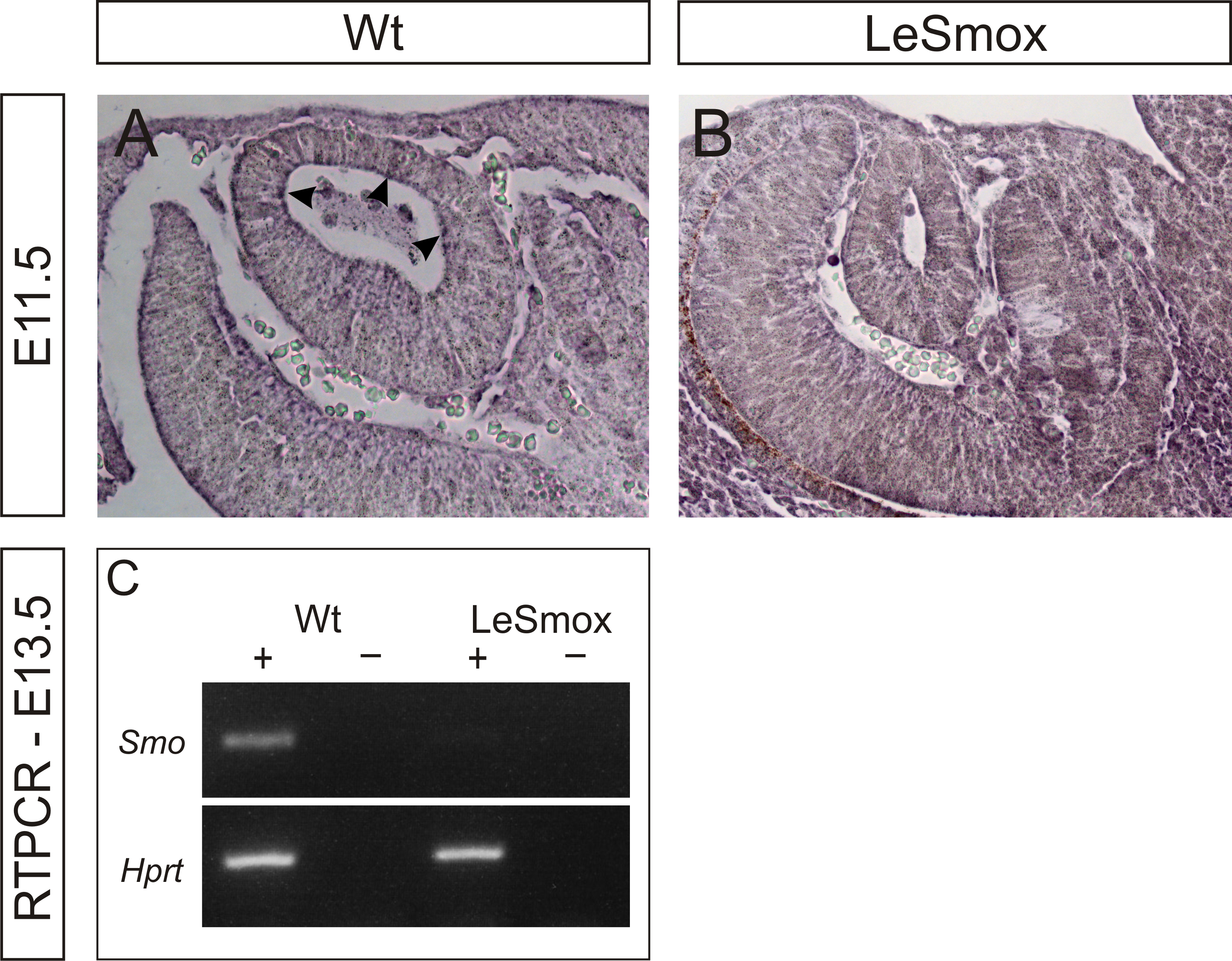

Supplement: Figure S3 — Loss of Smo expression in LeSmox lenses. In situ hybridisation for Smo mRNA in Wt (A) and LeSmox (B) lenses at E11.5 and RT-PCR (C) for Smo and Hprt in isolated E13.5 lenses, showing loss of Smo expression in LeSmox mutants. In Wt lenses at E11.5 (A), Smo mRNA is detected in the early lens pit/vesicle, particularly in the anterior vesicle cells (arrowheads) but also in the optic cup. B. In the LeSmox mutants at E11.5 signal for Smo mRNA is decreased in the lens pit. C. In the LeSmox mutants at E13.5, no expression of Smo is detected in isolated E13.5 lens vesicles by RT-PCR. Plus (+) and minus (−) signs indicate presence or absence of reverse transcriptase, respectively. Scale bar: 50 µm (A, B). (TIF) [file pone.0108037.s003.tif]
